# Present or absent: risks and protective factors of sudden infant death syndrome (SIDS) in the Zambian context

**DOI:** 10.7189/jogh.16.04273

**Published:** 2026-08-07

**Authors:** Ethan Zulu, Lawrence Mwananyanda, Rachel Pieciak, Leah Forman, Janaki Shah, Christopher J Gill, Roma Chilengi, Barbara Payne-Lohman, Cassandra Duffy, Godwin Osei-Poku, Donald Thea, Somwe Wa Somwe, Julie M Herlihy

**Affiliations:** 1 Right to Care Zambia, Lusaka, Zambia; 2 Department of Global Health, Boston University School of Public Health, Boston, USA; 3 Center for Health Data Science, Boston University School of Public Health, Boston, USA; 4 Department of Biostatistics, Boston University School of Public Health, Boston, USA; 5 Centre for Infectious Disease Research in Zambia, Lusaka, Zambia; 6 Institute for Immunology and Informatics, University of Rhode Island, Providence, USA; 7 Division of Maternal-Fetal Medicine, Department of Obstetrics & Gynecology, Beth Israel Deaconess Medical Center, Harvard Medical School, Boston, USA; 8 Division of Research and Analysis, Betsy Lehman Center for Patient Safety, Commonwealth of Massachusetts, Boston, USA; 9 Department of Pediatrics, Chobanian & Avedisian School of Medicine, Boston Medical Center, Boston University, Boston, USA; 10 Department of Pediatrics, School of Medicine, University of Zambia, Lusaka, Zambia

**Keywords:** sudden infant death syndrome, SIDS, SUID, infant sleep position, risk factors, sleep position

## Abstract

**Background:**

Despite a reduction in sudden unexplained infant death (SUID) in high-income countries, the incidence of SUID and the prevalence of its risk and protective factors remain poorly understood in Zambia due to limited research. We aimed to describe the infant sleep positions and sleep environments in an urban Zambian population to gain a better understanding of modifiable risk factors for SUID.

**Methods:**

We used antenatal, birth, and postnatal data from the Zambian Infant Cohort Study, a prospective birth cohort, to describe infant sleep practices in Chawama, a densely populated peri-urban community in Lusaka, Zambia. In addition to the longitudinal cohort data, we collected cross-sectional data on infant sleep practices and environmental risk factors associated with SUID during the 20-week study visit using a structured questionnaire.

**Results:**

We collected data from 596 caregivers and 605 infants. Overall, sudden infant death syndrome accounted for 8.3% (n/N = 3/36) of deceased infants. However, given the limited number of cases, this estimate should be interpreted with caution. Only 6.4% of caregivers attained an education beyond secondary school, and a significant proportion of infants (20.2%) had low birth weights, with 10.7% of infants confirmed by ultrasound as preterm. Furthermore, 96.5% of infants were placed to sleep on their side or in a prone position, and 98.2% of infants shared a sleep surface with their caregiver. Breastfeeding, a protective factor, reduced from 99.2% on day six to 43.1 at the 24-week visit.

**Conclusions:**

Both modifiable (bed-sharing and prone sleep position) and non-modifiable risk factors (low birthweight and prematurity) of SUID are prevalent in this low socioeconomic setting in Zambia. Public health strategies to prevent SUID need to be innovative and culturally congruent in addressing modifiable risks in settings where there is a lack of space.

Sudden infant death syndrome (SIDS) is the sudden death of an infant aged under one year without established cause after thorough investigation, including review of clinical history, circumstances of death, and performance of complete autopsy [1]. A recent postmortem surveillance study conducted in Zambia found that 7.4% of infant deaths in the community were due to sudden unexplained infant death (SUID), with nearly 5.4% attributed to apparent SIDS [2]. However, the prevalence of bed-sharing and prone or side sleeping positions, known risk factors for SIDS, is poorly understood in Zambia due to an apparent lack of research interest.

Deaths classified as SUID include SIDS and accidental suffocation and strangulation in bed. As a complex phenomenon, SIDS is characterised by multiple interacting risk factors. The triple risk hypothesis suggests that SIDS occurs in infants who have an underlying biological vulnerability and are exposed to an external threat during a critical developmental period. Numerous risk factors for SIDS are well documented globally, including low birth weight, male gender, young maternal age, and multiparity [3]. However, research on the prevalence of these non-modifiable risk factors in Zambia is limited to a single cross-sectional survey [4]. Additionally, several modifiable factors have been associated with a higher risk of SIDS, such as prone sleeping positions, bed-sharing, maternal smoking, and parental alcohol consumption [5]. In a recent qualitative study, Osei-Poku *et al.* [6] found that mothers viewed their baby's supine sleeping position as a choking hazard. They also preferred to bedshare with their infants for the convenience of breastfeeding and monitoring the baby. Debate persists regarding the potential benefits and risks of shared sleeping practices in relation to SIDS, and there has been little effort to examine the prevalence of these practices in the Zambian context.

Moreover, while a myriad of protective factors, including breastfeeding, pacifier use, and immunisation, have been associated with SIDS globally [7], no study in Zambia has comprehensively investigated them, partly due to the sluggish uptake and patchy nature of the investigative process surrounding SIDS in Zambia. We aim to build upon Osei-Poku’s cross-sectional study [4] by incorporating longitudinal and cross-sectional survey data, thereby mitigating some of the limitations of his earlier work.

## METHODS

### Population

We investigated infant sleep practices using a structured survey for mothers of 20-week-old infants participating in the Zambian Infant Cohort Study (ZICS). The ZICS is a longitudinal observational cohort consisting of 1500 mother-infant dyads in a peri-urban community in Lusaka, Zambia [8]. Briefly, from August 2019 to May 2022, ZICS enrolled pregnant women with and without HIV in a 1:1 ratio at their first antenatal visit and collected antenatal, birth, and postnatal data until six months postpartum. Pregnant women were recruited from the antenatal clinic at Chawama Hospital, a government health facility in Lusaka, Zambia. The ZICS staff comprised nurse midwives and physicians who provided maternal and child healthcare to dyads. The livebirths of these women defined the children who were HIV-exposed but uninfected and those who were not exposed to HIV. Trained midwives administered a structured questionnaire during the 20-week ZICS visit to gather data on the infants' sleeping environment and position from mothers enrolled in the birth cohort. A protocol amendment was only later approved to implement the structured sleep questionnaire when 596 mothers remained under follow-up in the ZICS study.

### Questionnaire development

We adapted the survey from the US Centers for Disease Control and Prevention’s revised 2020 Sudden Unexpected Infant Death Investigation Reporting Form and supplemented it with demographic questions from the ZICS cohort study. Subject matter experts in SUID, including Dr Julie Herlihy, contributed to the tool’s design. We culturally tailored the instrument for the Zambian context with input from local study staff. The questionnaire addressed infant sleeping practices, including bed-sharing, sleeping position, and bundling with clothing during sleep.

### Quality control

We electronically collected data regarding the infant sleep environment in a private room at the Chawama Hospital Maternal and Child Health Department. At the end of each day, the study coordinator (EZ) reviewed data collected by trained study staff. Additionally, data analysts (LF) conducted monthly data checks. We obtained written informed consent from every participant. For minors, written informed consent was obtained from their parents or guardians.

### Adjudication

We entered the abstracted infant death details into the database, including physical exam, history of present illness, radiology studies, laboratory results, and physician diagnoses. Before adjudication, we redacted HIV-related maternal or infant details. The adjudication panel consisting of two board-certified paediatricians (NN and JH) and one infectious disease physician (CG) jointly reviewed hospitalisations and deaths blinded to the child’s HIV-exposure status. The panel was asked to offer its best assessment of the primary syndromic diagnosis most likely to have caused the event (death) and whether the event was due to an infectious cause. If not enough data were available to determine diagnosis, the case was marked as ‘cause unknown’.

### Analytic method

We calculated descriptive statistics for the demographic characteristics of mothers and infants, as well as the SIDS risk factors related to the sleeping environment. We presented medians (interquartile ranges) for continuous variables and frequencies (percentages) for categorical variables. Additionally, we reported the numbers, percentages, and narratives of verbal autopsies linked to SUID.

We used SAS, version 9.4 (SAS Institute INC., Cary, USA) for all analyses.

## RESULTS

**Figure 1:**
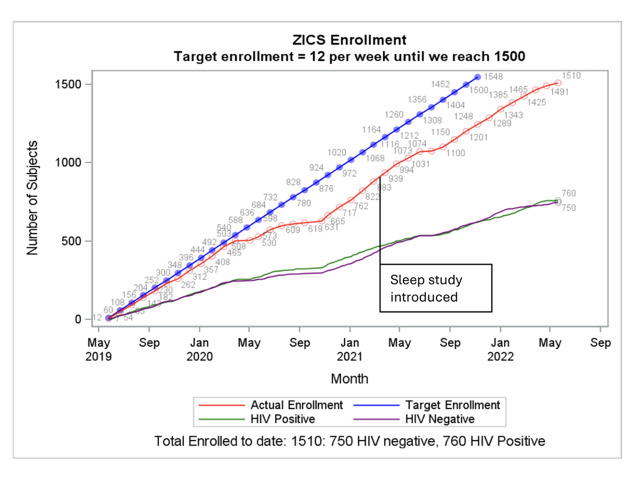
Timeline of the longitudinal and cross-sectional study. ZICS – Zambian Infant Cohort Study.

Data were collected from 596 caregivers both longitudinally and cross-sectionally (**Figure 1**). Most mothers were aged between 25 and 34 years (**Table 1**). Most mothers were married (n = 516; 86.6%), and only 6.4% attained an education beyond secondary school. Alcohol consumption during pregnancy was reported by 12.6% of the women, while 0.3% of women reported smoking during pregnancy. Of the 605 infants included in this study, 306 (50.6%) were male, and 299 (49.4%) were female (**Table 2**). Additionally, 65 (10.7%) respondents had preterm infants (<37 weeks gestation) confirmed by ultrasound, while 122 (20.2%) infants were born with low birth weight, and only 1 was born with a major abnormality. In our cohort, 43.1% of infants were exclusively breastfed at six months, and 90.3% had some breastfeeding at 6 months.

**Table 1:** Demographic characteristics of caregivers (n = 596)

	n (%)
**Age in years, x (SD)**	28 (6)
**Age group**	
17–24	172 (28.9)
25–34	319 (53.5)
≥35	105 (17.6)
**Marital status**	
Married	516 (86.6)
Not married	79 (13.3)
No answer	1 (0.2)
**Education**	
No formal education	13 (2.2)
Primary	145 (24.3)
Secondary	400 (67.1)
College (and above)	38 (6.4)
**Tobacco use**	
No	591 (99.3)
Yes, including during this pregnancy	2 (0.3)
Yes, but stopped in pregnancy	2 (0.3)
**Alcohol during pregnancy**	
No	520 (87.2)
Yes	75 (12.6)
No answer	1 (0.2)

SD – standard deviation, x̄ – mean

**Table 2:** Demographic characteristics of infants (n = 605)

	n (%)
**Sex**	
Male	306 (50.6)
Female	299 (49.4)
**Birthweight, g**	
>2500	478 (79.0)
1500–2500 (LBW)	122 (20.2)
1000–1500 (VLBW)	5 (0.8)
**Gestational week**	
Preterm (<37 weeks)	65 (10.7)
Term (37 completed weeks)	540 (89.3)
**Exclusive breastfeeding through 24 weeks**	
Labour	
Day 6	600 (99.2)
Week 6	584 (96.5)
Week 10	562 (92.9)
Week 14	520 (85.6)
Week 20	415 (68.6)
Week 24	261 (43.1)
**Any breastfeeding through 6 months**	
Labour	
Day 6	600 (99.2)
Week 6	595 (98.4)
Week 10	591 (97.7)
Week 14	587 (97.0)
Week 20	569 (94.1)
Week 24	546 (90.3)
**Major birth anomalies**	
No	604 (99.8)
Yes	1 (0.2)

LBW – low birth weight, VLBW – very low birth weight

Most infants (n = 593; 98.2%) shared a sleep surface with other adults, while a smaller number of babies (1.5%) had room sharing without bed-sharing (**Table 3**). Over three-quarters of babies were placed on their sides to sleep (n = 475; 78.5%), and less than a fifth (n = 109; 18%) of infants were placed in the prone position. A small proportion of infants (n = 21; 3.5%) were placed in the recommended supine or back sleeping position. When asked about the layers of clothing they use to wrap their babies, nearly three-quarters of infants (n = 44; 74.2%) were bundled in ≥2 layers.

**Table 3:** Risk factors for SIDS (n = 605)

	n (%)
**Child age in days at post-natal visit, x̄ (SD)**	148 (20)
**Child sleep position**	
On the infant’s side (lateral)	475 (78.5)
On the infant’s stomach (prone)	109 (18.0)
On the infant’s back (supine)	21 (3.5)
**Child sleep space**	
In a caregiver’s room on his/her own mattress or pad	9 (1.5)
In a caregiver’s bed with adult(s)	593 (98.2)
In another child’s room on his/her own mattress or pad	2 (0.3)
Child outfit in last two weeks*	
Just a nappy	54 (9.0)
One light layer of clothes	102 (16.9)
Two layers of clothes (for example with romper)	279 (46.3)
Two layers of clothes plus baby tightly swaddled in kitenge	53 (8.8)
Multiple layers including a blanket or shawl	115 (19.1)

SD – standard deviation, x̄ – mean

*The question asked: ‘Over the last two weeks, what type of outfit did you usually put your baby in for sleeping?’ The purpose was to assess the number of clothing layers or degree of bundling used.

During the study period, a total of 36 infants died. After careful adjudication of verbal autopsies and clinical case histories, SIDS accounted for 3 (8.3%) deaths among the deceased infants. Given the small number of cases, this estimate should be interpreted with caution. We collected a verbatim account of the narratives from two mothers whose babies died of SIDS. One mother stated, ‘It was shocking. It came so suddenly. We didn't see any signs’. Another mother said:


*I woke up to check and turn the baby around 01:00 on Monday (15/08/22), but to my surprise the baby was unresponsive, and blood was seen coming out from the nose and mouth with no pulse. We rushed to the hospital here at Chawama, and the baby was declared dead around 02:00.*

The third case was classified as SIDS during the adjudication panel review, and we did not conduct a verbal autopsy.

## DISCUSSION

The findings of this study, the first to incorporate both longitudinal and cross-sectional data on SIDS risk factors in Zambia, provide further evidence that both modifiable and non-modifiable risk factors for SIDS are highly prevalent in low socioeconomic settings. We found a SIDS prevalence of 8.3%, along with high rates of bed-sharing and lateral/prone sleeping positions, which are significant risk factors for SIDS.

Our results on infant sleeping positions appear consistent with other research that found a high prevalence of lateral and prone positions. These two positions are reported to carry the highest risk of SIDS among infants. Many mothers, however, believe that the prone and lateral positions prevent infants from accidentally aspirating their vomitus during sleep [6]. Our cohort also demonstrated a high uptake of breastfeeding. At six months, 43.1% of infants were reported to have exclusive breastfeeding, while 90.3% reported some form of breastfeeding. The extensive health benefits of breastfeeding for both mothers and infants have been widely reported [8]. While breastfeeding is protective against SIDS, it also promotes shared sleeping, as evidenced by the high percentage of infants (98.2%) with reported bed-sharing. Most researchers studying SIDS agree on the increased risk associated with bed-sharing, particularly in conjunction with prematurity and low birth weight [9]. On the other hand, proponents of co-sleeping argue that skin-to-skin contact (*i.e.* kangaroo care), especially among preterm and newborn infants, has empirical benefits. They contend that shared sleeping promotes thermoregulation, maternal-infant bonding, and a longer duration of breastfeeding [10]. This contradiction complicates health professionals' ability to offer the best possible advice to mothers.

Room sharing without bed-sharing is a recognised practice that significantly reduces SIDS. In contrast, only a very small proportion of mothers reported room sharing in our study. Our findings support previous observations indicating a low prevalence of room sharing without bed-sharing in these disadvantaged communities. Several factors could explain this observation. First, many of these families cannot afford a bed for their baby or a larger house due to their socioeconomic circumstances. Second, the culture of breastfeeding might reinforce bed-sharing practices over room sharing. Although little is known about how much clothing is required to maintain the infant’s thermal comfort, investigators generally agree that hyperthermia from overdressing should be avoided to reduce the risk of SIDS [11]. In our study, nearly three-quarters of mothers reported wrapping their babies in ≥2 layers of clothing. This finding aligns with Osei-Poku’s results [4], which indicated that several participants bundled their babies with at least two blankets during sleep.

Prematurity and low birth weight are well-known risk factors for SIDS. It has been suggested that the immature autonomic system leaves these infants vulnerable to an increased incidence of SIDS. In our study, a substantial percentage of infants (20.2%) were born with low birth weight. This figure is more than double the percentage of low birth weight recorded in Lusaka in the 2022 annual statistical report [12]. This difference can be partly attributed to the higher proportion of mothers living with HIV in our study sample (data not shown here), who are at an increased risk of delivering a low birth weight or premature baby [13]. This, combined with the high rates of bed-sharing, puts these infants at an increased risk of SIDS.

It is now well established from a variety of studies that maternal alcohol consumption and smoking increase the risk of SIDS [14]. While we found low levels of alcohol consumption and smoking in our cohort of mothers, we cannot conclude the prevalence of maternal smoking and alcohol consumption among SIDS mothers due to the scarcity of prospectively obtained SIDS incidences. A significant number of mothers in our study did not attain an education level beyond secondary school, which aligns with the rates reported by Osei-Poku *et al.* [4]. Given that most SIDS prevention campaigns focus on parental education and behavioural modification of infant care, this finding is noteworthy. Educational campaigns for SIDS in Zambia should be customised for mothers with low literacy [15].

### Limitations

Several limitations should be noted. First, we had to rely on self-reported data, particularly risk factors associated with infant care practices, which may be subject to social desirability bias. In addition, we were unable to confirm SUID cases using standard autopsy procedures and could not perform a sub-analysis examining the co-occurrence of SIDS risk factors among individual infants. However, a key strength of our study is its prospective cohort design, which enabled systematic data collection over time. For instance, we were able to obtain precise birth weight and gestational age by utilising accurate dating through ultrasound. Furthermore, we gathered more accurate breastfeeding patterns by administering a questionnaire during several routine visits.

## CONCLUSIONS

We investigated risk and protective factors of SIDS in a low socioeconomic setting. Our findings align with previous studies, which found that infant sleep and environment-related risk factors for SIDS are highly prevalent in disadvantaged communities. We also noted an increasing number of low birthweight and preterm infants who face a greater risk of SIDS in these vulnerable communities. Future public health promotion will need to address these challenges through carefully designed and targeted education for parents, grandparents, and healthcare providers. There is a need for a cross-national study encompassing both rural and urban areas, along with investigations into the co-occurrence of SIDS risk factors among individual infants. Our ongoing study on SIDS (Project Chisoni) already seeks to move beyond observational associations and toward stronger evidence that addresses issues of categorisation and causality.

## Data Availability

**Data availability:** Data that support the results of this study are available on the BEDAC repository and can be shared upon request.

## References

[1] Willinger M James LS Catz C Defining the sudden infant death syndrome (SIDS):deliberations of an expert panel convened by the National Institute of Child Health and Human Development. Pediatr Pathol. 1991;11:677–84. 10.3109/15513819109065465 1745639

[2] Osei-Poku GK Mwananyanda L Elliot PA MacLeod WB Somwe SW Pieciak RC The apparent burden of unexplained sudden infant deaths in Lusaka, Zambia: Findings from analysis of verbal autopsies. Gates Open Res. 2023;7:46. 10.12688/gatesopenres.14303.1

[3] Duncan JR, Byard RW. SIDS Sudden infant and early childhood death: The past, the present and the future. Adelaide, Australia: University of Adelaide Press; 2018. Available: https://digital.library.adelaide.edu.au/dspace/bitstream/2440/116995/2/hdl_116995.pdf. Accessed: 1 May 2026. 30024688

[4] Osei-Poku GK Mwananyanda L Elliot PA MacLeod WB Somwe SW Pieciak RC Assessing infant sleep practices and other risk factors of SIDS in Zambia: a cross-sectional survey of mothers in Lusaka, Zambia. BMC Pediatr. 2022;22:660. 10.1186/s12887-022-03712-5 36380292 PMC9664809

[5] Mitchell EA Thompson JM Zuccollo J MacFarlane M Taylor B Elder D The combination of bed sharing and maternal smoking leads to a greatly increased risk of sudden unexpected death in infancy: the New Zealand SUDI nationwide case control study. N Z Med J. 2017;130:52–64. 28571049

[6] Osei-Poku GK Mwananyanda L Elliott PA MacLeod WB Somwe SW Pieciak RC Qualitative assessment of infant sleep practices and other risk factors of sudden infant death syndrome (SIDS) among mothers in Lusaka, Zambia. BMC Pediatr. 2023;23:245. 10.1186/s12887-023-04051-9 37202764 PMC10193804

[7] Mitchell EA Krous HF Sudden unexpected death in infancy: a historical perspective. J Paediatr Child Health. 2015;51:108–12. 10.1111/jpc.12818 25586853

[8] Sharp M Campbell C Chiffings D Simmer K French N Improvement in long-term breastfeeding for very preterm infants. Breastfeed Med. 2015;10:145–9. 10.1089/bfm.2014.0117 25786115

[9] Matthijsse PR Semmekrot BA Liem KD [Skin to skin contact and breast-feeding after birth: not always without risk!]. Ned Tijdschr Geneeskd. 2016;160:D171. Dutch. 27484419

[10] Mekonnen AG Yehualashet SS Bayleyegn AD The effects of kangaroo mother care on the time to breastfeeding initiation among preterm and LBW infants: a meta-analysis of published studies. Int Breastfeed J. 2019;14:12. 10.1186/s13006-019-0206-0 30820239 PMC6379962

[11] Bach V Libert JP Hyperthermia and heat stress as risk factors for sudden infant death syndrome: A narrative review. Front Pediatr. 2022;10:816136. 10.3389/fped.2022.816136 35498814 PMC9051231

[12] Ministry of Health. Annual health statistical report. Lusaka, Zambia: Ministry of Health; 2022.

[13] Duffy CR Herlihy JM Zulu E Mwananyanda L Forman L Heeren T Preterm birth among women with HIV: impact of preconception cART initiation. AIDS. 2024;38:1749–57. 10.1097/QAD.0000000000003979 39017638 PMC11356690

[14] Hauck FR Blackstone SR Maternal smoking, alcohol and recreational drug use and the risk of SIDS among a US urban black population. Front Pediatr. 2022;10:809966. 10.3389/fped.2022.809966 35620144 PMC9127336

[15] Yamada MM Rosamilia MB Chiswell KE D’Ottavio A Spears T Osgood C Risk factors for sudden infant death in North Carolina. Front Pediatr. 2021;9:770803. 10.3389/fped.2021.770803 34956982 PMC8703192

